# Peptide Biomaterials for Tissue Regeneration

**DOI:** 10.3389/fbioe.2022.893936

**Published:** 2022-08-05

**Authors:** Alex Ross, Mildred A. Sauce-Guevara, Emilio I. Alarcon, Miguel A. Mendez-Rojas

**Affiliations:** ^1^ Division of Cardiac Surgery, University of Ottawa Heart Institute, Ottawa, ON, Canada; ^2^ Biochemistry, Microbiology and Immunology, University of Ottawa, Ottawa, ON, Canada; ^3^ Department of Chemical and Biological Sciences, Universidad de Las Américas Puebla, Puebla, Mexico

**Keywords:** peptides, bioactive materials, biomaterials, tissue regeneration, regenerative medicine

## Abstract

Expanding the toolbox of therapeutic materials for soft tissue and organ repair has become a critical component of tissue engineering. While animal- and plant-derived proteins are the foundation for developing biomimetic tissue constructs, using peptides as either constituents or frameworks for the materials has gained increasing momentum in recent years. This mini review discusses recent advances in peptide-based biomaterials’ design and application. We also discuss some of the future challenges posed and opportunities opened by peptide-based structures in the field of tissue engineering and regenerative medicine.

## 1 Introduction

Biomaterials have played various important roles in the field of tissue regeneration, from scaffolds that promote tissue regeneration to artificial skin and heart valve replacements (O'Brien, 2011). Biomaterials composed of biological or non-biological components have shown promise in preclinical studies (Gobi et al., 2021). Modification of the materials using peptide sequences can provide, for example, antihypertensive (Lu et al., 2014), antimicrobial (Annabi et al., 2017), angiogenic (Qu et al., 2018), anti-cancer (Matsuo et al., 2010; Almansour et al., 2012), neuronal growth (Hamsici et al., 2017; Cavanaugh et al., 2019), anti-apoptotic (Nam et al., 2019), drug delivery (Wei et al., 2017), and immunomodulatory properties (Korhonen and Pihlanto, 2006; Chen et al., 2019; Wang et al., 2020). Peptide functionalization can be achieved through chemical methods, such as hydrolysis, oxidation, grafting, and aminolysis, or through physical methods, such as adsorption, entrapment, formation of a self-assembled monolayer, or layer-by-layer deposition. The choice of method depends on the chemical composition of the nanomaterial and the peptide sequence (Holmes et al., 2015). In the following, we review selected examples of materials that have been chemically modified using peptides.

## 2 Naturally Occurring Materials

### 2.1 Alginate

**FIGURE 1:**
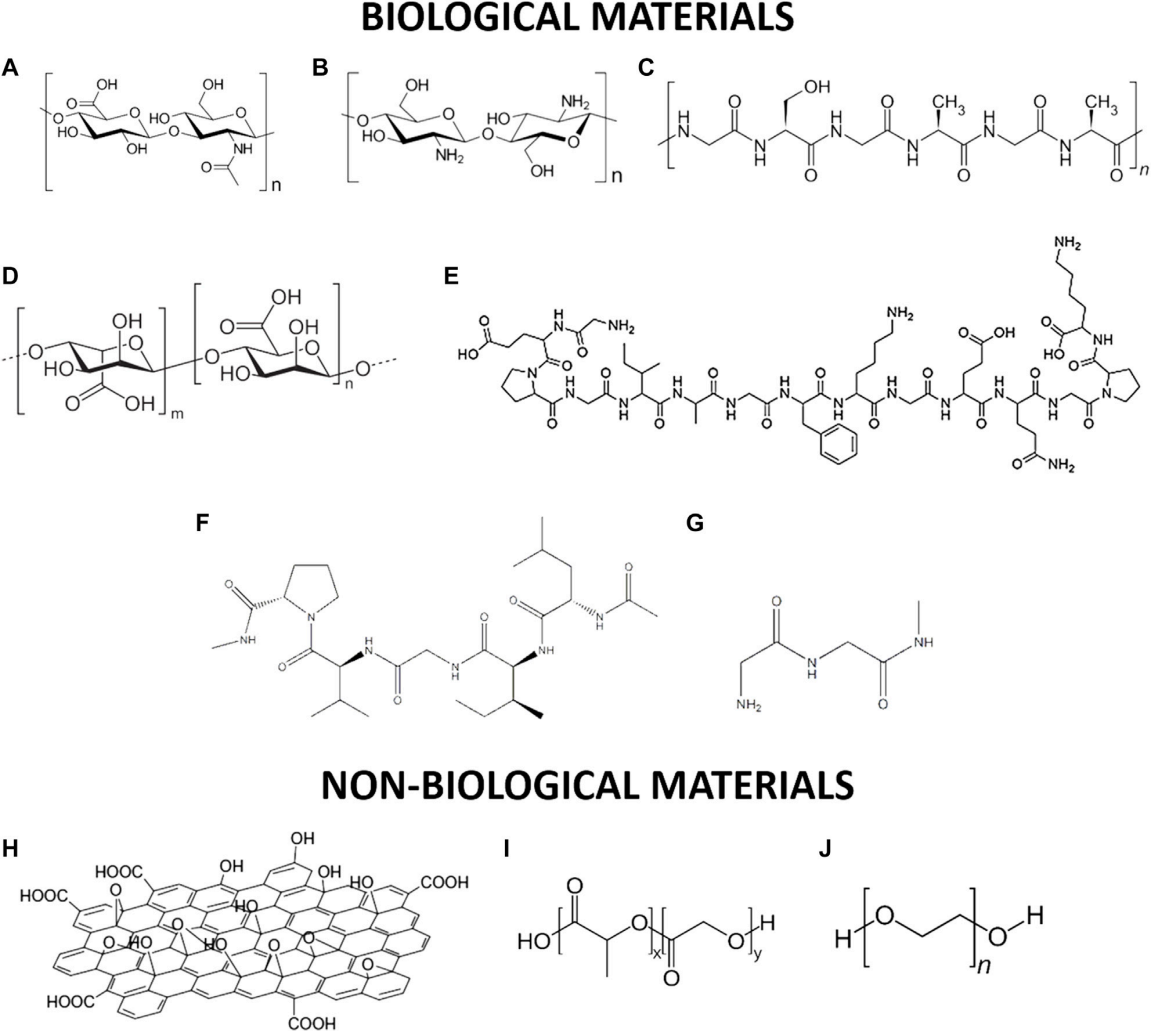
Schematic representation of selected biological and non-biological materials used for tissue regeneration: **(A)** alginate; **(B)** chitosan; **(C)** silk; **(D)** hyaluronic acid; **(E)** type II-collagen; **(F)** elastin; **(G)** fibrin; **(H)** graphene oxide; **(I)** poly(Lactide-co-Glycolide) **(J)** poly(ethylene glycol).

Alginate is a common biopolymer derived primarily from brown algae and bacteria (Lee and Mooney, 2012). It is a polysaccharide with ionic and hydrophobic properties, composed of two uronic acids: M (1–4)-linked β-D-mannuronic acid (M) and G α-l-guluronic acid (G) monomers (Sun and Tan, 2013) as showed in Figure 1. Alginate is non-immunogenic, biocompatible, and biodegradable (Angra et al., 2021). Its disadvantages include excessive water absorbability and low mechanical strength. Jay and Saltzman employed alginate microparticles to promote blood vessel formation and provide a sustained, localized release of heparin-binding growth factors, such as vascular endothelial growth factor (VEGF) (Jay and Saltzman, 2009).

### 2.2 Silk

Silk consists of 18 different amino acids organized into two groups of proteins, namely, fibroin and sericin (Wang et al., 2014a) represented in Figure 1. Silk is produced by larvae to form cocoons and the precise amino acid sequences used can vary from species to species. For instance, in the silkworm *Bombyx mori* (*B. mori*) the Gly-Ala-Gly-Ala-Gly-Ser hexapeptide sequence dominates the β sheet regions, while in *Antheraea pernyi* (*A. pernyi*) and *Philosamia ricini* (*P. ricini*), the polypeptide sequence is composed of stretches of polyalanine, resulting in a wide range of mechanical properties. Silk fibers have significantly better biocompatibility, degradability, strength, and durability than other materials (Kundu et al., 2014). Chen et al*.* made silk fibers by adding the RGD peptide sequence to improve the binding of human bone marrow stromal cells (Chen et al., 2003). Wenjie et al. used silk-based materials to promote rapid vascularization (Zhang et al., 2015). Park et al*.* reported the formation of a gel composed of silk and fibrin with hyaluronic acid for use as a scaffold in the formation of the cartilage of the nucleus pulposus, providing good biochemical support and conferring excellent mechanical properties (Park et al., 2011).

### 2.3 Collagen

Collagen is a structural protein that is responsible for strengthening the tendons, skin, and internal organs that make up about a quarter of the human body (Ricard-Blum, 2011). It is made up of three polypeptide chains wound together in a triple helix, with each of these chains being +1,400 amino acids long. In Figure 1 is shown the type II-collagen structure every third amino acid is glycine, and many of the remaining positions are made up of proline and hydroxyproline (Sorushanova et al., 2019). There are nearly 22 different types of collagens that exist in the human body with types I-IV being the most common. Huang et al. generated collagen nanofiber scaffolds for the repair of blood vessels and nerves using an electrospinning technique (Huang et al., 2011). Shen et al. developed a sponge composed of collagen and silk with the incorporation of exogenous SDF1α for the regeneration of ligaments and tendons (Shen et al., 2010). Yamauchi et al. prepared several layers of collagen and calcium phosphate that were mineralized by hydrolysis, resulting in the union and growth of fibroblast cells L929 (Yamauchi et al., 2004).

### 2.4 Elastin

Elastin (Figure 1) is a common extracellular matrix protein that is essential for the elasticity and resilience of many vertebrate tissues. Elastin’s precursor is tropoelastin which is associated with multiple molecules of elastin during elastogenesis to create a pattern of hydrophobic and hydrophilic sequences (Xiao et al., 2021) Represented in Figure 1. However, the insolubility of this protein presents a challenge for biomedical uses.

Flora et al. synthesized hydrogels based on elastin-like recombinamers with QK(KLTWQLYQLKYGIGI) and tested in human umbilical vein (HUVEC), where cell adhesion was shown; finally the injection of the hydrogel into a hind limb region in mice was demonstrated to enhance the formation of new capillaries within the constructs (Flora et al., 2019).

Buttafoco et al. developed collagen and elastin meshes using an electrospinning technique for the growth of smooth muscle cells (Buttafoco et al., 2006). Li et al. proposed a combination of elastin with collagen and poly(lactic-co-glycolic acid) (PLGA) to create scaffolds with good cytocompatibility towards both H9cr cardiac myoblasts and BMSC bone marrow stromal cells (Li et al., 2006). Wise et al. made a synthetic vascular conduit using cross-linked recombinant human tropoelastin with synthetic elastin fibers and polycaprolactone (Weisel et al., 2017).

### 2.5 Fibrin

Fibrin is a natural polymer essential for blood coagulation with a molecular weight of 360 kDa coming from the component fibrinopeptides showed in Figure 1 (Park et al., 2018). This material has been studied for decades for its use in tissue and organ repair (Shaikh et al., 2008). Bruns et al. developed a gel that functions as a fibrin-based matrix, which was delivered via intrahepatic injection for liver regeneration (Bruns et al., 2005). Chang et al. combined a fibrin-based glue with autogenous rabbit chondrocytes to create neocartilage through injection (Chang et al., 2007).

Losi et al. developed a poly(ether)urethane polydimethylsiloxane/fibrin-based scaffold loaded with PLGA nanoparticles containing the peptides VEGF and bFGF, which was tested in twenty-four male diabetic mice with an age of 10–11 weeks (Losi et al., 2013). Application of the scaffold to full-thickness dorsal skin wounds significantly accelerated wound closure after 15 days. This suggests complete re-epithelialization, with increased formation/maturity of granulation tissue and collagen deposition.

Gorodetsky et al. created fibrin-based microbeads of 50–200 μm that were tested to determine their binding to a wide range of cells. It was observed that binding of the hepatotactic cells to the fibrin-based microbeads led to a favorable wound-healing response (Gorodetsky et al., 1999).

### 2.6 Hyaluronic Acid

Hyaluronic acid (HA) is a non-sulfated GAG composed of repeating disaccharides of D-glucuronic acid and N-acetyl-D-glucosamine linked by a glucuronide bond (Papakonstantinou et al., 2012) represented in Figure 1. This material can be used as a sponge, hydrogel, or scaffold. For example, Chen et al. proposed the synthesis of an injectable biomimetic hydrogel based on hyaluronic acid-adipic dihydrazide and oxidized pectin loaded with an oligopeptide G4RGDS and tested in the presence of chondrocytes where good cell viability was shown (Chen et al., 2017). Additionally, the *in vivo* result confirmed that the hydrogel was tolerated within a mouse subcutaneous implantation model, which may result in a useful biomaterial for the regeneration of cartilage tissue.

Solchaga et al. generated sponges based on HYAFF11 and ACP; two biomaterials based on HA modified through esterification. These materials were tested as osteogenic or chondrogenic delivery vehicles, showing good compatibility for the cellular development of bone and cartilage (Solchaga et al., 1999). Likewise, Yang et al. made hydrogels of HA cross-linked with BDDE for application as an injectable scaffold to regenerate cartilage and dentin pulp complex using a preliminary subcutaneous microenvironment (Yang et al., 2016). Monteiro et al. synthesized a porous scaffold of HA through a layer by layer assembly technique assisted by spraying for the generation of a multilayer that promotes cell adhesion, contributing to the epidermal barrier of the skin (Monteiro et al., 2015).

### 2.7 Chitosan

Chitosan is a linear co-polymer containing D-glucosamine with N-acetyl-D glucosamine units linked by β-(1,4) glycosidic bonds, see Figure 1. The amino and hydroxyl groups can act as electron donors, and when the number of D-glucosamine units is high (≥50%), its solubility in acidic aqueous media increases. Another property this material presents is that different chemical modifications can be made to improve its intrinsic properties, such as thiolation, sulfonation, alkylation, and hydroxylation. Due to its antibacterial activity and minimal activation of the foreign body reaction, chitosan is often used for wound dressing applications (Muhsin et al., 2014).

Recently Ding et al. developed a hydrogel with the following biomaterials: chitosan, gelatin, and β-glycerophosphate, charged with the RGD peptide, and it showed improved *in situ* vascularization and further accelerating tissue repair/regeneration (Ding et al., 2020).

Chen et al. reported the preparation of a crosslinked chitosan-based asymmetric biodegradable membrane containing type I collage nanospheres and explored its use for wound healing (Chen et al., 2009). Mochizuki et al. developed chitosan-based membranes conjugated with peptides from laminin-1 (AGTFALRGDNPQG, RKRLQVQLSIRT) which were shown to promote cell attachment to actin stress fibers through interaction with an integrin receptor, leading to cell attachment along with proteoglycan-mediated filopodia formation (Mochizuki et al., 2003). Finally, Itoh et al. developed chitosan tubes coated with thiolated and non-thiolated hydroxyapatite conjugated with the CDPGYIGSR (YIGSR) peptide for application as nerve conduits. These tubes were grafted onto the sciatic nerve bridge of rats while muscle action potentials were recorded, giving rise to regenerated nerve tissue attached to thin layers of an epineurium-like structure (Itoh et al., 2005).

### 2.8 Graphene Oxide

Graphene oxide (GO) is composed of a hexagonal array containing two sub-networks of carbon atoms linked together by σ bonds. Each carbon atom has a π orbital that contributes to a delocalized network of electrons. This material has excellent physicochemical properties such as high intrinsic electron mobility, high Young’s modulus, and good thermal conductivity. GO can be synthesized either by Brodie or Hummer’s methods, both of which involve the oxidation of graphite to various levels (Zhu et al., 2010), see Figure 1. Eckhart et al. developed the functionalization of GO with the following peptides: polyglutamate (*p*(Glu)), which is known to promote osteogenesis, and polylysine (*p*(Lys)), which promotes cell adhesion and helps differentiation of stem cells into neuronal cells (Eckhart et al., 2019). Ahadian et al. prepared ultrathin films using a GO solution where their use as biocompatible substrates for the culture of C2C12 myoblast cells was evaluated (Ahadian et al., 2014). Finally, PLGA-based nanofiber plates co-functionalized with the RGD peptide and GO were manufactured by Shin et al. and their application to promote the union and proliferation of vascular smooth muscle cells (VSMCs) was evaluated, showing a favorable result for their use in VSMC regeneration (Shin et al., 2017).

### 2.9 Poly(Lactide-co-Glycolide)

PLGA is a biodegradable synthetic polymer that decomposes through hydrolysis into lactic acid and glycolic acid. It is a hydrophobic polymer that is easily eliminated in the body through the liver or spleen, see Figure 1. Due to this, it is coated with hydrophilic groups of poly(ethylene glycol) (PEG) to change its solubility. The use of this non-biological biomaterial in the biomedical area dates to the 1970s, when it was implemented in biodegradable sutures and implants (Essa et al., 2020). The newest suture application available on the market is Vicryl Rapide^®^, a modified version of the multifilament suture Vicryls^®^, which is a copolymer containing 90% glycolic acid (GA) and 10% l-lactic acid (LA). Bi et al. synthesized a scaffold based on PLGA and hydroxyapatite coated with collagen incorporating the peptide Asp-Gly-Glu-Ala (DGEA) for the repair of bone tissue in a rat cranial defect (Eckhart et al., 2019). Nune et al. produced aligned nanofibers through electrospinning of PLGA modified with RADA16-I-BMHP peptides which promoted greater expression and extension of Schwann cells, in addition to promoting the organization of native collagen, remyelination, and greater sensorimotor function in animal testing.

Nune et al. developed a method for the co-electrospinning of PLGA nanofibers with the peptide RADA16-I-BMHP1, showing its efficacy as a candidate for the efficient and functional regeneration of peripheral nerves since the results in Schwann cell adhesion increased significantly in the use of this material and the regeneration of the sciatic nerve in tests on rats showed favorable results (Nune et al., 2017). Kumbar et al. also developed matrices of PLGA through electrospinning for evaluation as skin substitutes, seeding them with human skin fibroblasts to achieve progressive growth (Kumbar et al., 2008).

### 2.10 Poly(Ethylene Glycol)

PEG is a neutral linear or branched polyether that is soluble in water and in most organic solvents. It repels other polymers in an aqueous environment, reflecting properties such as rejection of proteins, little immunogenicity, and formation of two phases with other polymers. PEG is non-toxic and can be chemically modified (Eckhart et al., 2019). PEG with molecular weights of less than 1000 has liquid, colorless and viscous properties, while PEG with higher molecular weights is a waxy, white solid. PEG is the most used biomaterial for the development of hydrogels and its structure is showed in Figure 1. Almany and Seliktar made a scaffold of fibrinogen cross-linked with PEG side chains to create a hydrogel that was adherent towards endothelial and smooth muscle cells (Almany and Seliktar, 2005). Yang et al. developed a scaffold based on PEG with the incorporation of the peptide Arg-Gly-Asp (RGD) that promotes osteogenesis of bone marrow stroma cells (Yang et al., 2005). Finally, Leslie-Barbick et al. used a peptide that mimics VEFG; Ac-KLTWQELYQLKYKGI-amide covalently bound to a PEG hydrogel matrix that promotes microvasculature coverage and improves vessel density and branching (Leslie-Barbick et al., 2011).

## 3 Peptides as Engineering Building Blocks in Therapeutics

### 3.1 Biofunctional Peptides

New peptides with biofunctional activity are constantly being discovered. For example, Song et al. used genomics to identify a short peptide from the skin of *Odorrana andersonii*, a species of frog from Asia, that may be used for wound healing (Song et al., 2019). *In vitro* data showed that treatment with the peptide increased the rate of keratinocyte repair during a scratch assay and reduced secretion of TNF and TGF-β1. *In vivo* data in a mouse wound healing model showed that topical application of an aqueous peptide solution increased the rate of wound healing. Fontoura et al. isolated the LPGPILSSFPQ peptide with antioxidant activity from keratin hydrolysis of feathers submerged in a *Chryseobacterium* sp. kr6 culture (Fontoura et al., 2019). Correa et al. showed that the MGTSSTDSQQAGHRRCSTSN peptide increases proliferation and leads to a mineralizing phenotype in human periodontal ligament cells (Correa et al., 2019). Ma et al. demonstrated that the peptide WQRPSSW inhibits angiogenesis and tumor growth (Ma et al., 2015). Bhatt et al. used C-peptide to reduce endothelial cell apoptosis under hyperglycemic conditions such as those found under uncontrolled diabetes (Bhatt et al., 2013). C-peptide reduced reactive oxygen species (ROS) generation through mechanisms including inhibition of protein kinase C and NADPH kinase. Cell viability was significantly improved by treatment with an aqueous 1 nM peptide solution down to levels approaching control viability, showing the potential of bioactive peptides to reduce cell apoptosis.

Peptides are also often used to induce stem cell differentiation. Lukasova et al. showed that the KIPKASSVPTELSAISTLYL peptide derived from the BMP-2 protein could affect mesenchymal stem cell (MSC) osteogenic differentiation (Lukasova et al., 2017). Kim et al. used self-assembling collagen mimetic peptides (CMPs) along with a poly(l-lactide-co-caprolactone) scaffold to cause chondrogenic differentiation in bone marrow stromal cells (Kim et al., 2015). Mohammed et al. used a self-assembling cadherin mimetic peptide hydrogel system to induce chondrogenic differentiation in human MSCs (Mohammed et al., 2021). Li et al. developed a hydrogel material for nerve tissue repair using HA and the peptide PPFLMLLKGSTR (Li et al., 2017). The base HA structure was prepared by cross-linking with adipic dihydrazide while peptides were added using ethyl N,N-dimethylaminopropyl carbodiimide and 1, 10-carbonyldiimidazole (CDI). The addition of peptide increased MSC spreading and growth while rat *in vivo* implantation of MSC containing HA-peptide hydrogels led to functional restoration of nerve tissue.

Along with inducing cellular differentiation, peptides can be also used to attract circulating cells into the material *in vivo*. Muylaert et al. created a ureido-pyrimidinone (UPy) polymer scaffold functionalized with the chemoattractant peptide SDF1α through electrospinning (Muylaert et al., 2016). Peptide functionalization increased cell migration numbers like the full-length SDF1α protein while decreasing inflammatory markers such as TNFα *in vitro*. An *in vivo* rat abdominal graft model showed increased cell number in peptide-functionalized gels along with deeper cellular penetration into the gels.

### 3.2 Material-Peptide Combinations

Bioactive peptides are often used to biofunctionalize otherwise inert materials for use in cell culture (Table 1). Gill et al. modified a synthetic PEG with RGD adhesion peptide and GGGPQGIWGQGK (PQ) matrix metalloproteinase (MMP)-sensitive peptide (Gill et al., 2012). Lung adenocarcinoma cells were encapsulated in the functionalized synthetic hydrogels and cell morphology matched that seen in Matrigel extracted animal protein gels. Furthermore, matrix stiffness and peptide concentration were found to affect cell morphology. Synthetic peptides can be used to tune the mechanical properties of materials based on animal-derived proteins. Gouveia et al. combined a collagen gel with the self-assembling amphiphilic Fmoc-RGDS adhesion peptide (Gouveia et al., 2014). These combined gels were stronger and more stable than the collagen gels alone. Furthermore, functionalization improved the cell viability of human corneal stromal fibroblasts cultured on functionalized gels over collagen gels alone. To reduce peptide degradation by native proteases and improve cell internalization. Nanoparticles can be used. Sun et al. loaded antimicrobial CATH30 peptides through adsorption into carboxymethyl chitosan nanoparticles to use in wound healing (Sun et al., 2018). *In vitro* data indicated that the nanoparticles released their peptide payload over 12 h and caused increased keratinocyte cell migration in a scratch assay. *In vivo* data in a mouse wound model indicated that the peptide nanoparticles accelerated wound healing and led to a much smoother regrown dermal layer.

**TABLE 1:** Summary of peptide biomaterials.

Peptide name (sequence)	Polymer Used	Experimental	Results
Vascular endothelial growth factor (VEGF) (Jay and Zaltzman 2009)	Alginate microparticles crosslinked by CaCl_2_, ZnCl_2_, and SrCl_2_.	Development and synthesis of small alginate microparticles (<10 μm mean diameter) by using different ionic crosslinkers.	The cross-inked particles allowed the sustained release of bioactive VEGF and showed no toxicity in HUVECs
RGD peptide (Arginine-Glycine-Aspartic acid) (Chen et al., 2003)	Silk fibers	Modified silk matrices were prepared by covalent coupling of RGD peptides and compared to matrices without this coupling. An MTT assay was performed with BMSC and ACLF cell lines seeded on both matrices for 14 days.	Modification of silk fibroin fiber matrices with RGD-binding sites significantly enhanced attachment and spreading in BMSC/ACLF cells while no effect on cell proliferation was observed.
SDF1α (Shen et al., 2010)	Silk-collagen sponge	In 14 female rats weighing 200–220 g, a 6 mm portion of their Achilles tendon was removed from their left limb to implant the control scaffold (woven silk and collagen sponge + collagen gel containing PBS). The right limbs were implanted with a composite peptide scaffold (silk and collagen woven sponge + collagen gel containing SDF1α).	After 4 weeks, the SDF1α -treated tendon had increased expression of tendon repair genetic markers and endogenous SDF1α, exhibited more physiological microstructures with larger diameter collagen fibrils, and had better biomechanical properties than the control group.
CMPs KLD12:Ac-KLDLKLDLKLDL-NH_2_ KLD12-CMP7:Ac-KLDLKLDLKLDLGGPOGPOGPOGPOGPOGPOG-POG-NH_2_ (Kim et al., 2015)	Poly(l-lactide-co-caprolactone)	Hydrogel complexes were cultured in a chondrogenic medium and real-time PCR evaluation was performed to assess chondrogenic differentiation. Hydrogels were implanted into the subcutaneous dorsum of mice with *in situ* chondrogenesis and cartilage tissue formation analysis after 5 weeks.	CMP motifs were shown to significantly increase gene expression related to chondrogenic differentiation.
PPFLMLLKGSTR [(Li et al., 2017)] Motif sequence derived from laminin-5 α3 chain	Hyaluronic acid	A hyaluronic acid hydrogel scaffold was prepared by cross-linking with adipic dihydrazide while adding peptides using ethyl N,N-dimethylaminopropyl carbodiimide and CDI for use in spinal cord tissue restoration. The hydrogel was tested on MSCs to investigate cell viability, and SD female rats weighing 220–250 g were used for *in vivo* tests. The scaffolds were spinal cord-shaped, 1.5 mm thick, and a laminectomy was performed exposing the dorsal surface of the T9-10 segment.	The addition of peptide increased MSC spreading and growth while rat *in vivo* implantation of MSC containing HA-peptide hydrogels led to functional restoration of nerve tissue.
SDF1α (R y NR) (Muylaert et al., 2016)	Poly(l-lactide-co-caprolactone) functionalized with quadruple hydrogen bonding ureido-pyrimidinone (UPy) units	Eighteen male Sprague Dawley rats weighing between 350 and 450 g received an electrospun aortic interposition graft. Grafts were explanted on day 1 or day 7. Electrospun tubular scaffolds were engrafted in rat abdominal aortas and explanted for histological analysis after 24 h and 7 days.	Modification of poly(l-lactide-co-caprolactone) with UPy and SDF1α peptides helped retain and stimulate circulating cells to improve the cellularization of implanted vascular replacement grafts.
RGD and PQ: GGGPQGIWGQGK (Zhu et al., 2010)	Poly(ethylene glycol)	344SQ cells from KRasG12D/p53R172HΔG mice were encapsulated in hydrogels with varying concentrations of PEG-PQ [5%, 10% or 15% (w/v)] and PEG-RGDS (1, 3.5, or 7 mmol/L). Immunohistochemistry tests and quantitative RT-PC were performed to assess the response to TGF-β.	Cell-adhesive PEG-RGDs with an enzyme-degradable PEG-PQ backbone were able to induce MET in 344SQ cells to form lumenized polarized spheres and also repressed miR- 200 after TGF-β exposure with concomitant change in EMT marker gene expression.
YIGSR, RGD and REDV (Ahadian et al., 2014)	Silk fibroin scaffolds	Silk fibroin scaffolds were functionalized with peptides. Platelet adhesion and activation was assessed along with HUVEC adhesion, proliferation, and migration.	Scaffolds modified with dual peptides (YIGSR + RGD) significantly improved HUVEC proliferation and also had an increased effect on cell migration relative to scaffolds modified with only individual peptides.
RDG and GHK Ada-Ahx-GGRGD and Ada-Ahx-GGGHK (Shin et al., 2017)	Poly(hydroxyethyl methacrylate)	Cryogels were synthesized by vinyl addition polymerization in aqueous solution and functionalized with peptide and tested with PC-12 rat pheochromocytoma cells and NIH 3T3 mouse embryonic fibroblast cells.	Poly(hydroxyethyl methacrylate) synthetic cryogels functionalized with peptides provided a controllable/stable charge and high specific activity in the tested cell lines in addition to showing a synergistic effect on cell proliferation in 3T3 and PC-12 cells.
OH-CATH30 (OH30) (Essa et al., 2020)	Carboxymethyl chitosan nanoparticles	Nanoparticles were synthesized from carboxymethyl chitosan and loaded with CATH30 antimicrobial peptides. OH30 release behavior of nanoparticles in simulated wound fluid (SWF) was assessed along with antibacterial activity. Cell migration assays were performed on the HaCaT cell line. Evaluation and measurement of wound healing was carried out in female mice of 6 weeks of age and average weight 20–25 g. Injured with a skin biopsy punch (ID = 7 mm).	Wound healing in the OH-CATH30 group was significantly accelerated compared to the administration of CMCS or OH30 alone. Expression of anti-inflammatory cytokines was increased along with an improvement in cell migration. No effects on keratinocyte metabolism and proliferation were observed.
GRGDYP, GRGDSP KHIFSDDSSE (Almany and Seliktar, 2005)	Alginate	Peptide-coupled calcium alginate hydrogels were synthesized by partial oxidation with periodate followed by reductive amination. Adhesion tests were performed on C2C12-type mouse skeletal myoblast cells and human dental (RP89) stem cells.	While C2C12 myoblasts adhered to both functionalized and control gels, RP89 cells only adhered to alginate gels with the highest concentrations of peptide.
RGD (phenol2-poly(ethylene glycol)-RGD) (Almany and Seliktar, 2005)	Hyaluronic acid-tyramine	Horseradish peroxidase and hydrogen peroxide were used to simultaneously crosslink the HA gel and incorporate phenol-containing peptides. The gel was subcutaneously injected in a 6- to 9-week-old mouse model along with HUVECs and human fibroblasts.	HUVECs cultured on or within the RGD-modified hydrogels showed adhesion behavior that led to enhanced cell proliferation, migration, and formation of a capillary-like network. When HUVECs and human fibroblasts (HFF1) were encapsulated together in the RGD-modified hydrogel, functional vasculature was demonstrated within the cell-laden gel after 2 weeks in subcutaneous tissue.
YIGSR QK: VEGF (Song et al., 2019)	Poly(ethylene glycol) and gelatin	Poly(ethylene glycol) and gelatin hydrogels were functionalized with peptides and tested on human umbilical vein endothelial cells containing 2% fetal bovine protein (FBS) and growth factors with the exception of serum VEGF. VEGFR2/KDR phosphorylation, gene expression and immunofluorescence assays were carried out.	The inclusion of QK, a VEGF-mimetic peptide, led to a strong biological response to *in vitro* gels in HUVEC cells, as measured by an increase in phosphorylated VEGFR2 and a change in cell morphology.
CGGRGDS (Gentsch et al., 2011)	Poly(lactide-co-glycolide)	The synthesis of nanofiber meshes was carried out in a one-step process resulting in surface biofunctionalization with peptide segments. The fiber surface was assayed with photoelectron spectroscopy (XPS) and adhesion data were collected by mapping forces at the apex of fibers on a grid. L929 murine fluorescent renal fibroblasts were used for adhesion and migration tests.	Bioavailability and bioactivity of peptides on fiber surfaces was demonstrated, resulting in meshes promoting increased fibroblast adhesion and migration compared to pure PLGA meshes.
Peptide amphiphiles (PAs) YIGSR KKKKK MMP-2-sensitive sequences GTAGLIGQ (Bhatt et al., 2013)	Polycaprolactone	Polycaprolactone nanofibers were electrospun along with peptide amphiphiles. SEM and TEM were used to characterize the morphology of the nanofibers and confirm the coating of PA on the surface of the ePCL nanofibers. Nanofibers were tested on HUVECs and human aortic smooth muscle cells (AoSMC) to assess cellular adhesion and proliferation.	The hybrid nanomatrix of self-assembling PA-coated ePCL nanofibers provided stimulative mechanical strength and topographic structure, promoting endothelial cell-specific increased adhesion and proliferation while limiting smooth muscle cell proliferation.
Derived from the laminin B1: TS(CDPGYIGSRAS)_8_ Derived from fibronectin: TS(CDPGYIGSRAS)_8_ and (TGRGDSPAS)_8_ (Gouveia et al., 2014)	Silk fibroin	Tests of resistance to breakage of WT and recombinant silk fibers were carried out along with an *in vivo* long-term safety evaluation where four sponges of 5 mm in diameter and 2.5 mm in thickness were implanted in the paravertebral muscle in male SD rats with a body weight of 200–280 g and 8 weeks of age. Cell adhesion migration assays were performed on Balb/c 3T3 mouse fibroblasts.	Recombinant silk fibroin films incorporating only the L- or H-chain-independent TS sequence (CDPGYIGSRAS)_8_ showing significantly increased adhesive activities in mouse endothelial and smooth muscle cells in addition to high migration activities of endothelial cells.
Angiopoietin-1 derived peptide: QHREDGS (Reis et al., 2012)	Chitosan-collagen	Conjugation of QHREDGS to chitosan was carried out using 1-ethyl-3-(3-dimethylaminopropyl)carbodiimide HCl (EDC) chemistry, then an evaluation of peptide conjugation efficiency was made. Subsequently, the chitosan and collagen hydrogel was synthesized and SEM, degradation, and rheological tests were carried out. An MI mouse model was used in adult male C57 Black-6 mice.	Subcutaneous injection of peptide-functionalized hydrogel in rats had the ability to localize to the injection site and retain cells, with CM contractile apparatus identified after 7 days.
Laminin peptide (YIGSR) (Itoh et al. 2005)	Chitosan and Hydroxyapatite	Chitosan tubes from crab tendon coated with hydroxyapatite were functionalized with YIGSR peptide. In male Sprague-Dawley (SD) rats weighing 180 g, the right sciatic nerve of each rat was exposed and a 10 mm long section was excised. The synthesized tube was grafted onto the nerve and electrophysiological and histological evaluations were carried out.	YIGSR-conjugated tube transplantation resulted in regenerated nerve tissue attached to thin layers of epineurium-like structure formed on the surface of the inner tube, enhancing axonal nerve regeneration and promoting proximal nerve stump and bridge sprouting.
Laminin peptide AG73: RKR-LQVQLSIRT (Monteiro et al., 2015)	Chitosan	The preparation of a chitosan membrane was carried out with the peptide CGGRKRLQVQLSIRT. Human newborn foreskin keratinocytes were used for adhesion and propagation tests. In 8-week-old 22–26 g BALB/c Slc-nu male nude mice, the fasciae of the abdominal muscles was exposed and an AG73 chitosan membrane was placed.	The AG73 peptide-conjugated chitosan membrane promotes cell adhesion and propagation *in vitro*, with 80% of human keratinocytes adhering to membranes within 2 h. *In vivo*, application of the membrane established a stratified epidermis-like structure in the fascia.
Polyglutamate (*p*(Glu)) Polylysine (*p*(Lys)) (Eckhart et al., 2019)	Graphene oxide	Synthesis of NCA monomers was carried out to carry out the electrophilic synthesis of CG (ECG), later the synthesis of the peptides was carried out by means of encapping. Subsequently, dispersions of CG and *p*(Lys)long–G (0.5 mg mL^−1^ in deionized water) were prepared and their pH was adjusted. Cell culture was carried out on PCL12 cells, a cell line that was isolated from a rat pheochromocytoma.	Functionalization of graphene oxide with conductive and biocompatible peptides was carried out to make three-dimensional mechanically robust constructions. The conductivity and bioactivity of these Pep-G materials was demonstrated by electrically stimulated PC12 cells cultured on a *p*(Lys) long-G pellet showing enhanced neurite adhesion and growth.
VEGF15: Ac-KVKFMDVYQRSYCHP-amide QK: Ac-KLTWQELYQLKYKGI-amide (Leslie-Barbick et al., 2011)	Poly(ethylene glycol)	The synthesis of hydrogels based on polyethylene glycol was carried out by preparing and purifying different combinations of this material with the peptides QK, RGDS and VGEF. The bioactivity assay was carried out in human umbilical vein endothelial cells and finally the *in vivo* assay was performed in Flk1-myr::mCherry transgenic mice.	In response to the QK-peptide functionalized hydrogel, endothelial cells formed tubule structures and established cellular connections. *In vivo* results showed a more complete coverage of the host microvasculature within the hydrogel, as well as improvement in the points of contact, branching, and density of blood vessels.
OA-GL12 GLLSGINAEWPC (Kumbar et al., 2008)	Not applicable	Peptides were synthesized through Fmoc-SPPS and tested in keratinocytes (HaCaT), human skin fibroblasts (HSF), human umbilical vein endothelial cells (HUVEC), and murine macrophages. In adult male mice weighing 22–25 g, full-thickness skin wound models were made after 7 days of acclimatization.	Peptide treatment resulted in an improvement in wound healing. The secretion of tumor necrosis factor (TNF) and transforming growth factor β1 (TGF-β1) in the murine macrophage cell line was decreased. Histological analysis indicated that mice treated with OA-GL12 (10 nM) displayed increased regeneration of neo-epidermis and restoration of the dermis.

While most materials utilize a single bioactive peptide for a singular purpose such as cell adhesion, the combination of multiple peptides with different bioactivities may also show beneficial effects. Peng et al. electrospun silk fibroin matrices functionalized with multiple peptides (Peng et al., 2019). Surface functionalization was performed using carbodiimide chemistry after electrospinning. Certain combinations of peptides such as YIGSR + RGD had a combinatorial effect on HUVEC cell proliferation, indicating that multiple peptides could be used to maximize desired biological responses in specific situations. Luong et al. created poly(hydroxyethyl methacrylate) cryogels to show that the peptides RDG and GHK exhibit a synergistic effect in terms of increasing cell proliferation in 3T3 and PC-12 cells (Luong et al., 2020).

It has been shown that the covalent and selective functionalization of materials with bioactive peptides can become favorable around tissue engineering by providing cell attachment to biomaterials through biochemical signals and interaction with membrane receptors (Brun et al., 2021).

Peptides can be covalently attached to nanomaterials for purposes such as facilitating delivery. Pantarotto et al. covalently conjugated the α_s_ subunit of the G_s_ protein in peptide form to a carbon nanotube (CNT) (Pantarotto et al., 2004). For covalent conjugation, maleimide groups were added to the CNTs which reacted with a terminal cysteine group added to the peptide. These CNT-peptide structures were shown to translocate across the cell membrane and efficiently enter the cell, offering an effective method of delivery into the cell. Thornton and Heise used amine-functionalized silica nanoparticles capped with Fmoc-peptide acting as a gating agent (Thornton and Heise, 2010). Sequence-specific cleavage of the gate peptide by enzymes such as thermolysin or elastase led to release of molecular payload from the nanoparticle. This enzyme-mediated release (EMR) platform may allow for the targeting of specific tissues where certain enzymes are upregulated.

### 3.3 Peptide Coupling Methods

**FIGURE 2:**
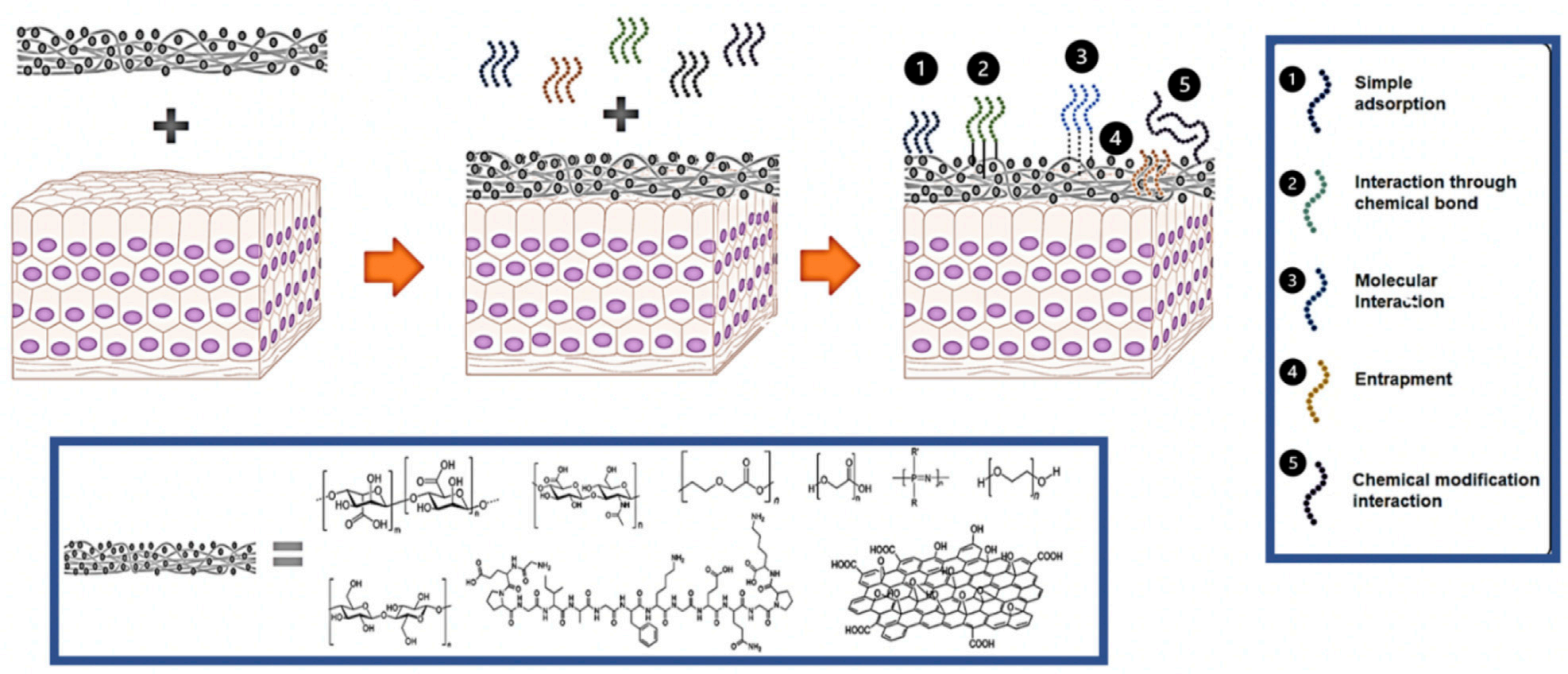
Schematic representation for the interactions between materials and peptides. Peptides can be added to a biomaterial through 1) simple adsorption, 2) covalent conjugation, 3) molecular interactions, 4) entrapment, or through 5) chemical modifications.

Peptides are often covalently coupled to scaffold materials such as alginate, gelatin, HA, PLGA, and many others (Figure 2). While carbodiimide chemistry is commonly used for this coupling, Dalheim et al. developed a two-step periodate oxidation to reductive amination method for coupling of peptides to alginate (Dalheim et al., 2016). Higher substitution densities that those commonly obtained with carbodiimide chemistry were achieved with no detectable by-products. These higher densities were required for RP89 cells to effectively adhere to the material. Another method for covalent modification of polysaccharides uses thiol-ene chemistry. Mergy et al. developed a two-step method esterification of hydroxyl groups performed using pentenoic anhydride followed by reaction with a mercaptan under ultraviolet light (Mergy et al., 2012). The water-soluble photoinitiator Irgacure 2959 was used to generate a thiyl radical, resulting in a rapid (5 min) and efficient aqueous reaction that could be used to attach peptides. A dithiol PEG polymer was used in this reaction as a Dextran crosslinking agent, resulting in a hydrogel whose viscosity could be tuned by modifying the polysaccharide concentration. Wang et al. used a one-step enzyme-mediated methodology to covalently conjugate HA to peptides containing phenols (Wang et al., 2014b). Horseradish peroxidase and hydrogen peroxide were used to simultaneously crosslink the HA gel and incorporate phenol-containing peptides. This gel, when injected *in vivo* along with HUVECs and human fibroblasts subcutaneously in a mouse model, led to the observation of functional vasculature after 2 weeks. Su et al. used a PEG succinimidyl valerate crosslinker, otherwise known as PEGX, to create a hydrogel out of gelatin and biofunctional peptides (Su et al., 2019). PEGX reacts with the free amines found in both gelatin and peptides, crosslinking them. The inclusion of QK, a VEGF mimetic peptide, led to a strong biological response to the gels *in vitro* in HUVEC cells, as measured by an increase in phosphorylated VEGFR2 and a change in cell morphology. Electrospinning is often used to incorporate peptides into a biomaterial scaffold. Gentsch et al. developed a single step process to generate PLGA fibers that are surface functionalized with peptide using electrospinning (Gentsch et al., 2011). By conjugating the CGGRGDS peptide to PLLA and then electrospinning a solution containing PLGA and the PLLA-peptide conjugate, nanofibers could be made with strong expression of the peptide at their surface where it can interact with cells. Adding methanol to the chloroform used in 1:3 ratio allowed the authors to change the diameter of the fibers.

### 3.4 Non-covalent Functionalization Methods

In addition to covalent chemical coupling, peptides can be attached to a material through non-covalent methods (Figure 2). These methods include adsorption, molecular interactions, and entrapment. Use of non-covalent interactions is especially desirable when the peptide is intended to be released from the material after delivery. The physical process of adsorption represents the simplest way to incorporate peptides into a material. This process depends on the affinity between a surface and peptide to determine how strong the adsorption interaction will be. Micksch et al. screened different peptides for their ability to bind to zirconium oxide (ZrO2), titanium zircon (TiZr), and titanium oxide (TiO2), materials commonly used in medical implants (Micksch et al., 2014). Some of the peptides showed almost no adsorption, but certain sequences could attach to each surface. In addition to peptide sequence, post-translational modifications can affect a peptide’s affinity for a given surface. For example, Xiaoqing et al. showed that phosphorylation improved adsorption of peptide onto a hydroxyapatite surface (Liu et al., 2011).

In addition to adsorption, molecular interactions between peptides and materials can facilitate peptide incorporation. Ananthanarayanan et al. used molecular interactions to add bioactive peptide amphiphiles into supported phospholipid bilayers through vesicle fusion (Ananthanarayanan et al., 2010). The bilayers adsorbed onto a glass surface and vesicles containing up to 40% peptide could be formed. Culturing NSCs on the bilayers showed that peptide incorporation improved cell adhesion. Ge et al. combined the β-hairpin peptide CBHH with succinic, malic, and tartaric dicarboxylates to induce peptide self-assembly and subsequent gelation (Ge et al., 2021). Addition of the dicarboxylates improved the cell compatibility of the peptide while allowing it to form a gel. Electrostatic interactions are also commonly used to functionalize a material. Aye et al. added the cationic antimicrobial peptide polymyxin B to anionic silk fibroin to create an antimicrobial hydrogel (Aye et al., 2022). Increasing peptide concentration altered the optical densities of the gels and increased their zeta potentials. Diffusion tests indicated that the gels containing polymyxin B had antimicrobial activity towards *E. coli* and *A. baumannii*.

Finally, entrapment, the physical enclosure of peptides by a surrounding material, can sometimes be used to provide peptide functionalization. Bauer et al. entrapped a mixture of random antimicrobial peptides in a copper metal matrix through solution ion reduction. Incorporation of peptide in the copper matrix led to a lower material density and smaller particles. The peptide-material showed synergistic antimicrobial activity against MRSA with the composite materials showing a greater inhibition than the sum of the copper and peptide used alone.

### 3.5 Supramolecular Peptide Structures

Peptides can also be assembled into structures without covalent bonding using supramolecular interactions. Niece et al. created a charged peptide amphiphile (PA) system consisting of two separate positively and negatively charged peptides that self-assemble upon mixing in aqueous solution at physiological pH (Niece et al., 2003). Transmission electron microscopy (TEM) revealed that the peptides formed long nanofibers where the hydrophobic tails were likely hidden in the center of the fibers as oppositely charged peptides came together to achieve stability via salt-bridging.

On the other hand, Tomas et al. developed an amyloid-inspired PA self-assembled to furnish kinetically controlled nanofibers and incorporated in a dynamic covalently cross-linked polysaccharide network of carboxymethyl cellulose dialdehyde and carboxymethyl chitosan (CMCh) using Schiff base chemistry, where the non-covalent interaction provided the mechanical properties to the hydrogel developed for its application in chondrogenesis, where an improved cell growth for the *in vitro* testing was demonstrated (Thomas et al., 2021).

Webber et al. created a PA system with 10% bioactive RGD peptides and 90% diluent peptides and used the system for cell encapsulation (Webber et al., 2010). Encapsulated cells were found to be viable and proliferative, with a 5.5-fold growth after 5 days. Subcutaneous injection of the PA system with encapsulated cells resulted in 3.2-fold increased bioluminescent signal at day 4 over baseline, indicating cell survival and proliferation *in vivo*. Berns et al. modified a PA platform to display the bioactive peptide epitopes IKVAV and RGDS (Berns et al., 2014). These self-assembling peptides formed aligned nanofibers in solution, as seen with TEM. The culture of neurons in gels formed by the addition of CaCl_2_ to peptide solution led to increased neurite growth and the detection of spontaneous action potentials. Standley et al. modified a PA system with the (KLAKLAK)_2_ peptide, which can induce cell death via membrane disruption (Standley et al., 2010). Addition of the KLAK PAs to breast cancer cell culture resulted in a 50% reduction in cell viability. Ceylan et al. created a PA system consisting of dopa (3,4-dihydroxyphenyl-l-alanine) conjugated peptides and REDV epitope conjugated peptides to bind onto stainless steel surfaces commonly used in stents (Ceylan et al., 2011). Dopa binds to both organic and inorganic surfaces, and thus the inclusion of dopa in the PA system lead to nanofibers that were effectively attached to the steel surface. The REDV epitope selectively binds epithelial cells, leading to effective cell binding to the peptide-treated surface. Thota et al. created a peptide assembly system to produce a hydrogel for wound dressing (Thota et al., 2020). The short peptide hydrogelators using the nonproteinogenic amino acid α,β-dehydrophenylalanine (ΔPhe) in LΔF and the fMLF isoniazid and the antibiotics ciprofloxacin and amphotericin B. A gel was successfully made that slowly released the antibiotics over time and showed good cytocompatibility.

### 3.6 Peptide Materials in Cardiovascular Repair

Due to their bioactive characteristics, peptides are routinely incorporated into biomaterial scaffolds used for repairing cardiovascular tissues. Andukuri et al. developed a matrix for cardiac repair using electrospun polycaprolactone (ePCL) nanofibers, along with self-assembled PAs (Andukuri et al., 2011). The ePCL provided a porous base structure while the peptide YIGSR adhesive ligands and KKKKK nitric oxide donors provided biofunctionality. HUVEC and smooth muscle cells grown on the matrix showed increased proliferation. Rufaiha et al. developed a self-assembling glycosaminoglycan (GAG) mimetic peptide nanofiber scaffold for use in cardiac tissue repair after myocardial infarction (MI) (Rufaihah et al., 2017). These nanofibers were shown to promote cardiomyocyte adhesion and proliferation *in vitro*. After injection *in vivo*, an increase in neovascularization along with improved functional cardiac performance was seen, indicating the potential use of these materials for cardiac repair. Tongers et al. used a bioactive peptide matrix to support bone marrow-derived pro-angiogenic cells (BMPACs) for use in ischemic tissue repair (Tongers et al., 2014). The peptide matrix was composed of a self-assembling PA system displaying the RGDS adhesion ligand. *In vitro* data indicated that peptide matrices reduced cell apoptosis and increased cell number during BMPAC culture. *In vivo* delivery of BMPACs in peptide matrix led to increased functional recovery and perfusion. To develop a material that can be used as a vascular graft, Asakura et al. modified recombinant silk fibroin with the TS(CDPGYIGSRAS)_8_ peptide (Asakura et al., 2014). Peptide-functionalized silk fibroins showed increased cell adhesion and migration by vascular endothelial TDK2 cells. Vascular graft implantation in an *in vivo* rat model showed an increase in cell migration distance into the gel in peptide-functionalized gels. Jha et al. generated a HA-based hydrogel functionalized with CGGNGEPRGDTYRAY peptide and heparin that were crosslinked by a bis-cysteine MMP-degradable peptide (Jha et al., 2015). *In vitro*, the gels increased cardiac progenitor cell (CPC) adhesion and proliferation while also increasing the amount of angiogenic cytokines released by the cells. *In vivo*, the gels enhanced CPC survival and increased angiogenesis in a subcutaneous mouse model.

Peptides alone can also create a restorative effect in cardiac tissue. Yasuda et al. treated ischemia/reperfusion (I/R) injury with T3 peptide (Yasuda et al., 2019). *In vitro* data indicated that the addition of T3 peptide inhibited oxygen and glucose deprivation followed by reoxygenation reduced H9c2 cardiomyoblast apoptosis in a dose-dependent manner. *In vivo* data in a rat MI model showed that treatment with peptide decreased infarcted heart area and reduced the loss of left ventricular developed pressure (LVDP) following MI. Wu et al. used lipopolysaccharide (LPS) to induce acute lung injury (ALI) in a mouse model and then treated the resulting injury with hydrostatin-SN1 (H-SN1) (Wu et al., 2017). Treatment with H-SN1 peptide reduced lung tissue permeability and reduced the number of cells present in the bronchial alveolar lavage fluid. *In vitro*, LPS-stimulated RAW 264.7 cells released less inflammatory cytokines after treatment with H-SN1, indicating the potential use of the peptide in healing lung injuries.

Pulmonary delivery of peptide materials provides an alternative route to injection. Tewes et al. co-spray dried salmon calcitonin peptide along with PEG and PVP to create aerosoliable particles. Particle properties could be adjusted by varying the solvent and PEG/PVP ratios. These particles elicited a strong biological response as measured by cAMP production by T47D cells (Tewes et al., 2010).

Peptides can also be used to coat implanted devices for improved biocompatibility. Kushwaha et al. created a self-assembled PA peptide matrix for the coating of cardiovascular diseases (Kushwaha et al., 2010). As nitric oxide (NO) is thought to reduce platelet adhesion which can lead to clotting while increasing endothelial cell proliferation, the C_16_-GTAGLIGQKKKKK peptide was used as a NO source while C_16_-GTAGLIGQYIGSR was used as a cell adhesion ligand selective to endothelial cells. This peptide system shown *in vitro* to increase HUVEC proliferation and spreading while reducing platelet adhesion to values below that of stainless steel. Reis et al. created a composite hydrogel from chitosan and type I-collagen functionalized with the angiopoietin-1 peptide QHREDGS (Reis et al., 2012). Peptide addition did not change the hydrogel’s mechanical properties but did increase encapsulated cardiomyocyte cell metabolism and the proportion of gels exhibiting beating behavior increased. Subcutaneous *in vivo* injection of this matrix in a rat model showed increased myofibroblast and cardiomyocyte number at the injection site in the peptide-functionalized gel.

### 3.6 Other Medical Applications

In addition to their application in repairing cardiovascular tissues, peptide materials are used for the repair of many other tissue types. Among these, the repair of damaged bone tissue is a common goal for biomaterials application. Kolambkar et al. developed a hybrid system consisting of an electrospun nanofiber mesh combined with peptide-modified alginate hydrogel to deliver recombinant bone morphogenetic protein-2 for the repair of large segmental bone defects (Kolambkar et al., 2011). Growth factor was quickly released from the material, with 99% of release occurring within 7 days. The material was also shown to significantly improve bone function after 12 weeks in rats, with extracted femora displaying increased maximum torque and torsional stiffness. Huang et al. functionalized a poly(lactide-co-glycolide)/nano-hydroxyapatite surface by using polyethyleneimine to introduce active groups onto the surface (Huang et al., 2010). Functionalization of the surface with peptide improved the ability of BMSCs to adhere and resulted in increased cell proliferation after 7 days. Implantation of the material in a preliminary rabbit mandibular defect model showed improved bone regeneration after 1 week. Mata et al. generated self-assembled peptide amphiphiles containing phosphoserine residues to help promote bone regeneration (Mata et al., 2010). Upon addition of CaCl_2_ to the dissolved peptides, β-sheet formation led to solution gelation. The gels were implanted into fractured bone in a rat model with the peptide treatment group possessing a larger bone volume after 4 weeks. Ko et al. explored a novel surface functionalization method by using dopamine polymerization under alkaline conditions to coat PLGA scaffolds with bone morphogenetic protein-2 derived peptides (Ko et al., 2013). The peptides were well-attached to the PLGA surface with 85–90% of peptide remaining after 6 days of incubation. The materials were then used to induce osteogenic differentiation of human adipose-derived stem cells and implanted into calvarial bone defects, leading to improved bone regeneration.

In addition to bone regeneration, peptide materials are often applied towards skin repair where they can enhance wound healing and help fight infection. Ej et al. used PVA nanofibers to deliver nanoparticles of microRNA-31 encapsulated in CHAT peptide to a mouse skin wound model (Ej et al., 2022). Due to the cell penetrating CHAT peptides, a transfection efficiency of 15% was achieved in HaCaT keratinocyte cells. When the material was applied *in vivo*, epidermal thickness was increased 7 days after treatment. Chen et al. used SIKVAV peptide modified chitosan hydrogels to affect inflammatory cytokines in a mouse skin wound model (Chen et al., 2018). The material successfully lowered the expression of the IL-1β, TNF-α and IL-6 inflammatory cytokines at 3, 5, and 7 days after application. Furthermore, the material promoted angiogenesis, keratinocyte proliferation, and collagen synthesis while reducing the wound healing time. Carrejo et al. used a multidomain peptide to produce a hydrogel that is easily infiltrated by cells for treating a diabetic mouse skin wound model (Carrejo et al., 2018). NIH-3T3 fibroblasts readily spread and proliferated through the gels in culture so that cell networks were formed within the gel by 7 days. When applied *in vivo*, greater skin regeneration was observed, along with increased blood vessel density.

### 3.7 Outlook

The last 50 years of research has opened the door to the development and use of biomaterials at both pre-clinical and clinical level for treating damaged tissues. However, most of those biomaterials rely on the use of naturally extracted polymers, including animal proteins, which present additional challenges to the already complicated process of clinical translation of biomaterials. Bioinspired synthetic peptides present a cost-effective way to provide, or boost, functional properties to otherwise inert materials. Thus, for example, using a combination of peptides and standard biopolymers can render materials with improved functionality, biocompatibility, and durability. While surface modification or loading of peptides within the materials remain the most used approaches, advancements in peptide computational design that includes the ability to predict folding and supramolecular assembly together with the use of bio-orthogonal chemistry bear the promise of developing the next generation of peptide-based materials as alternatives to naturally derived polymers. Those materials will have impact beyond the regenerative medicine realm, where for example, their use as bioinks for 3D bioprinting presents some very attractive areas for peptide-based biomaterials.
